# m^6^A RNA Methylation Regulators Act as Potential Prognostic Biomarkers in Lung Adenocarcinoma

**DOI:** 10.3389/fgene.2021.622233

**Published:** 2021-02-10

**Authors:** Hongbo Wang, Xiangxuan Zhao, Zaiming Lu

**Affiliations:** Department of Radiology, Shengjing Hospital of China Medical University, Shenyang, China

**Keywords:** lung cancer, N6-methylAdenosine (m^6^A), prognosis, epitranscriptomics, cancer biomarker

## Abstract

N^6^-methyladenosine [m(6)A/m^6^A] methylation is one of the most common RNA modifications in eukaryotic cell mRNA and plays an important regulatory role in mRNA metabolism, splicing, translocation, stability, and translation. Previous studies have demonstrated that the m^6^A modification is highly associated with tumor cell proliferation, migration, and invasion. In the present study, five m^6^A regulatory factors have been revealed, namely heterogeneous nuclear ribonucleoprotein A2/B1(HNRNPA2B1), heterogeneous nuclear ribonucleoprotein C (HNRNPC), Vir like m^6^A methyltransferase associated protein (KIAA1429/VIRMA), RNA binding motif protein 15 (RBM15) and methyltransferase like 3 (METTL3), which are closely related to the overall survival (OS) of patients with lung adenocarcinoma (LUAD). These five m^6^A regulatory factors exhibited potential prognostic value for the 1, 3, and 5-years survival outcomes of LUAD patients. Our findings revealed that several signaling pathways, such as cell cycle, DNA replication, RNA degradation, RNA polymerase, nucleotide excision repair and basal transcription factors, are activated in the high-risk group of LUAD patients.

## Introduction

Lung cancer is currently one of the most common malignant tumors presenting the highest fatality rate among all malignancies (Siegel et al., 2020). In opposite to the surgical resection of early lung cancer, advanced lung cancer is mainly treated with radiotherapy and/or chemotherapy, while adjuvant immunotherapy and targeted therapy are also administered (Hirsch et al., 2017). Most patients have already advanced lung cancer by the time of their diagnosis mainly due to the limited knowledge in the pathogenesis of lung cancer, and the 5-year survival rate does not exceed 20% (Siegel et al., 2020). The clinical prognosis of patients is mainly based on tumor stage and other clinical indicators such as tumor node metastasis (TNM) stage. However, huge variation is observed in the final prognosis of the same tumor stage as a result of patients' heterogeneity. Therefore, relying on simple tumor staging may lead to poor prognosis accuracy, greatly affecting patients' further treatment and reducing the overall survival rate (OSR) (Razzouk, 2014; Perakis et al., 2016). The identification of accurate prognostic markers can contribute to the improvement of the treatment of lung cancer patients.

N^6^-methyladenosine (m^6^A) refers to the N^6^ terminal methylation of adenosine, which is a ubiquitous post-transcriptional modification mechanism of RNA in eukaryotic cells (Chen et al., 2019). m^6^A is involved in the RNA metabolism, and more specifically in mRNA translation, degradation, splicing, export, and folding (Liu et al., 2017; Chen et al., 2019; Liu and Gregory, 2019). The completion of m^6^A modification requires the binding of methyltransferase with the conservative motif RRACH (R=A/G, H=U/A/C) in RNA (Kane and Beemon, 1985; Narayan et al., 1994; Balacco and Soller, 2019). m^6^A often occurs in the stop codons of the 3′ untranslated (3′UTRs) and exon regions, respectively (Dominissini et al., 2012; Meyer et al., 2012). m^6^A modification is usually a reversible process that is regulated by various related factors (Jia et al., 2011, 2013). The m^6^A regulators reported so far can be divided into three types. The first type is called Writers including METTL3, METTL14, METTL16, WTAP, KIAA1429, RBM15, and ZC3H13, which are able to recognize RNA and modify m^6^A (Dai et al., 2018; Balacco and Soller, 2019). The second type is Erasers that include fat mass- and obesity-associated protein (FTO) and alk B homolog 5 (ALKBH5). These regulators are mainly responsible for removing m^6^A modifications (Liu et al., 2018; Pan et al., 2018). The third type is Readers that consist of YTHDF1, YTHDF2, YTHDF3, YTHDC1, YTHDC2, HNRNPC, and HNRNPA2B1. Readers can recognize RNA methylation modifications and further regulate RNA processing, translation, and degradation (Wang et al., 2018; Ma et al., 2019).

Functional analysis has shown that m^6^A is crucial for cell proliferation, cell self-renewal, and apoptosis as it affects many important life processes (Zhou et al., 2019). A large number of studies have confirmed that the aberrant m^6^A modification plays a key role in the occurrence and progression of various tumors including LUAD (Zhou et al., 2019; Yi et al., 2020; Zhang et al., 2020). For instance, m^6^A Reader YTHDF2 can promote the non-small cell lung cancer (NSCLC) progression (Sheng et al., 2019), while the Eraser ALKBH5 can inhibit the metastasis of NSCLC by inhibiting the miR-107/LATS2-mediated YAP activity (Jin et al., 2020). In addition, m^6^A status can also affect the sensitivity of NSCLC to Afatinib treatment (Meng et al., 2020). However, the potential value of m^6^A for the prognosis of lung cancer treatment still remains unexplored, especially for the prognosis of LUAD. The present study initially confirmed that the expression levels of five m^6^A regulators, including HNRNPA2B1, HNRNPC, KIAA1429, RBM15, and METTL3 were correlated with OS of LUAD patients. m^6^A Writers regulatory factors were also suggested as potential prognostic biomarkers for LUAD.

## Materials and Methods

### Data Acquisition

The LUAD gene expression data and the corresponding clinical data were downloaded from The Cancer Genome Atlas database (TCGA) (*https://cancergenome.nih.gov/)* by using TCGA-assembler in February 2020^1^. Gene expression in the downloaded files was normalized using the Fragments Per Kilobase of exon model per Million mapped fragments (FPKM) metric. Data for the m^6^A regulators including METTL3, METTL14, METTL16, WTAP, KIAA1429, RBM15, ZC3H13, FTO, ALKBH5, YTHDF1, YTHDF2, YTHDF3, YTHDC1, YTHDC2, HNRNPC, and HNRNPA2B1, were retrieved by mining the transcriptomics data of LUAD and para-carcinoma tissues. The human tissue expression levels in Genotype-tissue expression (GTEx) database were downloaded in May 2020. The GTEx dataset contains more than 900 organs and tissues of healthy people, with a total of more than 17,000 samples, covering 54 types of tissues in the human body.

### Bioinformatics Analysis

Gene expression data of the tumor and control sample were separately sorted. Gene expression data for the 16 m^6^A related genes were then extracted and data with incomplete information were deleted. R software (Version 3.6.1) was used to perform differential expression analysis on the m^6^A regulatory factors in lung tissue samples in a comparison between 497 LUAD tissues (from 467 LUAD patients) and 54 para-carcinoma tissues. Vioplot tool was used to plot violin graphs to visualize the results of the differential expression analysis between LUAD patients and control samples. The Spearman correlation analysis was deployed to study the associations between the expression levels of the 16 m^6^A-related regulatory factors.

The Least Absolute Shrinkage and Selection Operator (LASSO) model is a dimensionality reduction method, which can reduce the number of variables through a penalty mechanism and ultimately achieve the goal of reducing bias. The LASSO model analysis was performed using the glmnet R package. Gene Set Enrichment Analysis (GSEA) software (Version 4.0.3 from Broad Institute official website homepage) was used to analyze the enrichment analysis between high-risk and low-risk groups. A total of 55,268 genes were included in the analysis. Moreover, the “c2.cp.kegg.V7.0.symbols.gmt” analysis package was used to study the pathway enrichment. All the LUAD samples were divided into two groups (high-risk and low-risk groups) by median risk score. P-value and False Discovery Rate (FDR) thresholds of 0.05 and 0.25, respectively, were used to infer significant findings.

### Statistical Analysis

One-way analysis of variance (Anova) test was used to compare the 16 m^6^A regulatory factors in between 497 LUAD tissues and 54 para-carcinoma tissues. The Spearman correlation was used to clarify the relationship between the m^6^A gene expression level and the basic clinical information (such as age, gender, TMN stage) of LUAD patients. The OSR is defined as the time period from diagnosis to death. Univariate and multivariate COX logistic regression models were used to analyze the prognostic potential of each factor and their ability to predict the survival outcome of LUAD. Kaplan-Meier and receiver operating characteristic (ROC) curves were plotted to demonstrate the prognostic performance of the explored m^6^A related regulators. A prognostic model was established by drawing a Nomogram plot, and calibration curves were used to verify it. P-value threshold of 0.05 was used to infer statistical significance.

## Results

### m^6^A Regulator mRNA Levels

**Figure 1:**
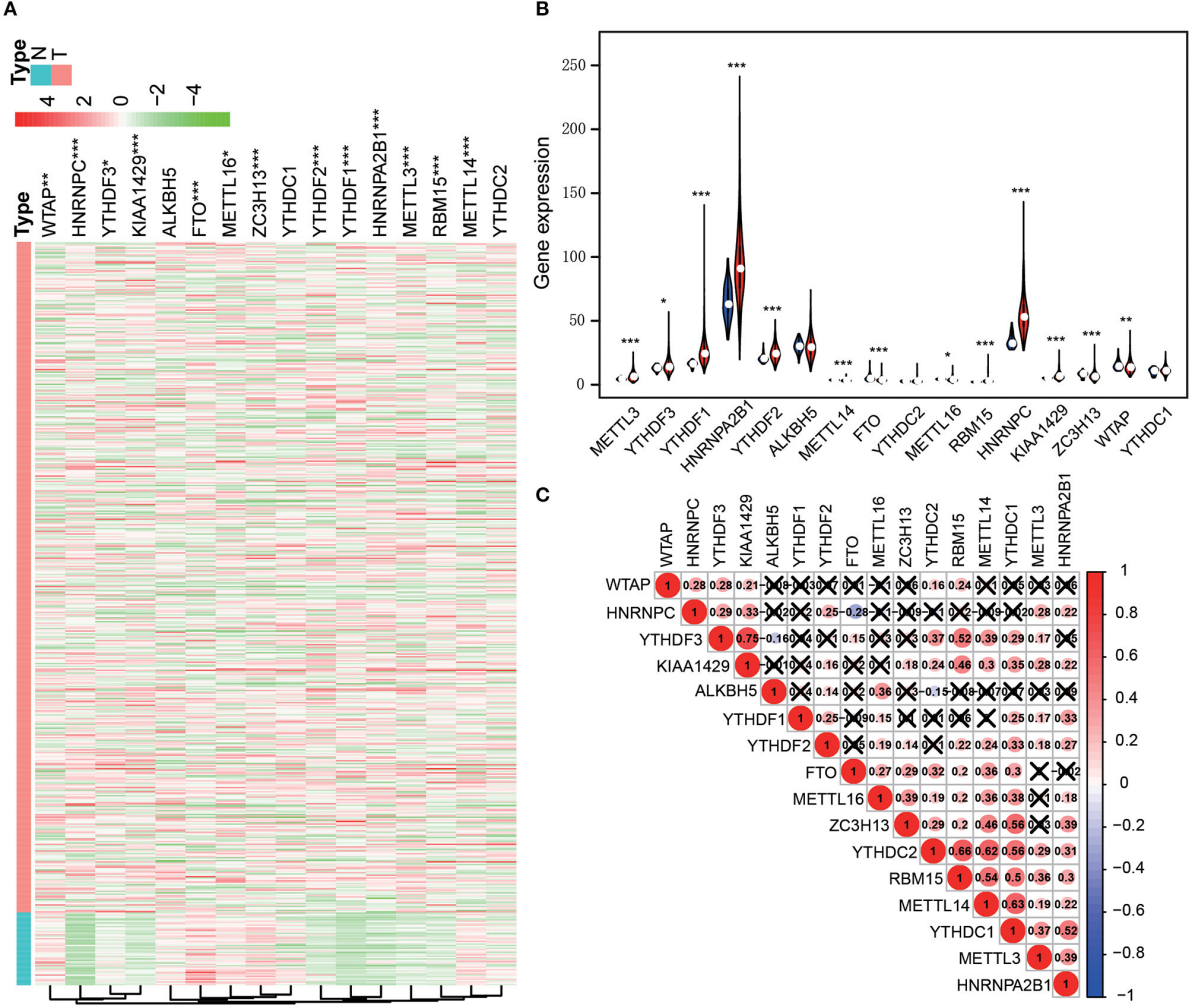
The 16 m^6^A-related genes were differentially expressed in the comparison of LUAD with normal tissues. **(A)** Heatmap (***p < 0.001; **p < 0.01; *p < 0.05; Red means high expression and green means low expression). **(B)** Violet plots (***p < 0.001; **p < 0.01; *p < 0.05; Blue represents normal tissue and red represents cancer tissue). **(C)** The correlation between the expression levels of each m^6^A-related gene.

After screening the mRNA expression levels of the m^6^A regulators in 497 LUAD and 54 normal control samples, respectively, we analyzed the expressions of 16 m^6^A related regulators: METTL3, METTL14, METTL16, WTAP, KIAA1429, RBM15, ZC3H13, FTO, ALKBH5, YTHDF1, YTHDF2, YTHDF3, YTHDC1, YTHDC2, HNRNPC and HNRNPA2B1. Among them, METTL3, METTL14, KIAA1429, RBM15, ZC3H13, FTO, YTHDF1, YTHDF2, HNRNPC, HNRNPA2B1, WTAP, YTHDF3 and METTL16, were significantly overexpressed in LUAD tissues. The expression of ALKBH5, YTHDC1, and YTHDC2 presented no statistically significant differences (Figures 1A,B). Moreover, Pearson correlation analysis was conducted between the 16 m^6^A related regulators. We found that the positive correlation between the expression levels of YTHDF3 and KIAA1429 was the highest one. Furthermore, significant positive correlations were revealed between YTHDC1, YTHDC2, RBM15, and METTL14 from the same analysis (Figure 1C).

### m^6^A-Related Gene Expression and LUAD Prognosis

**Figure 2:**
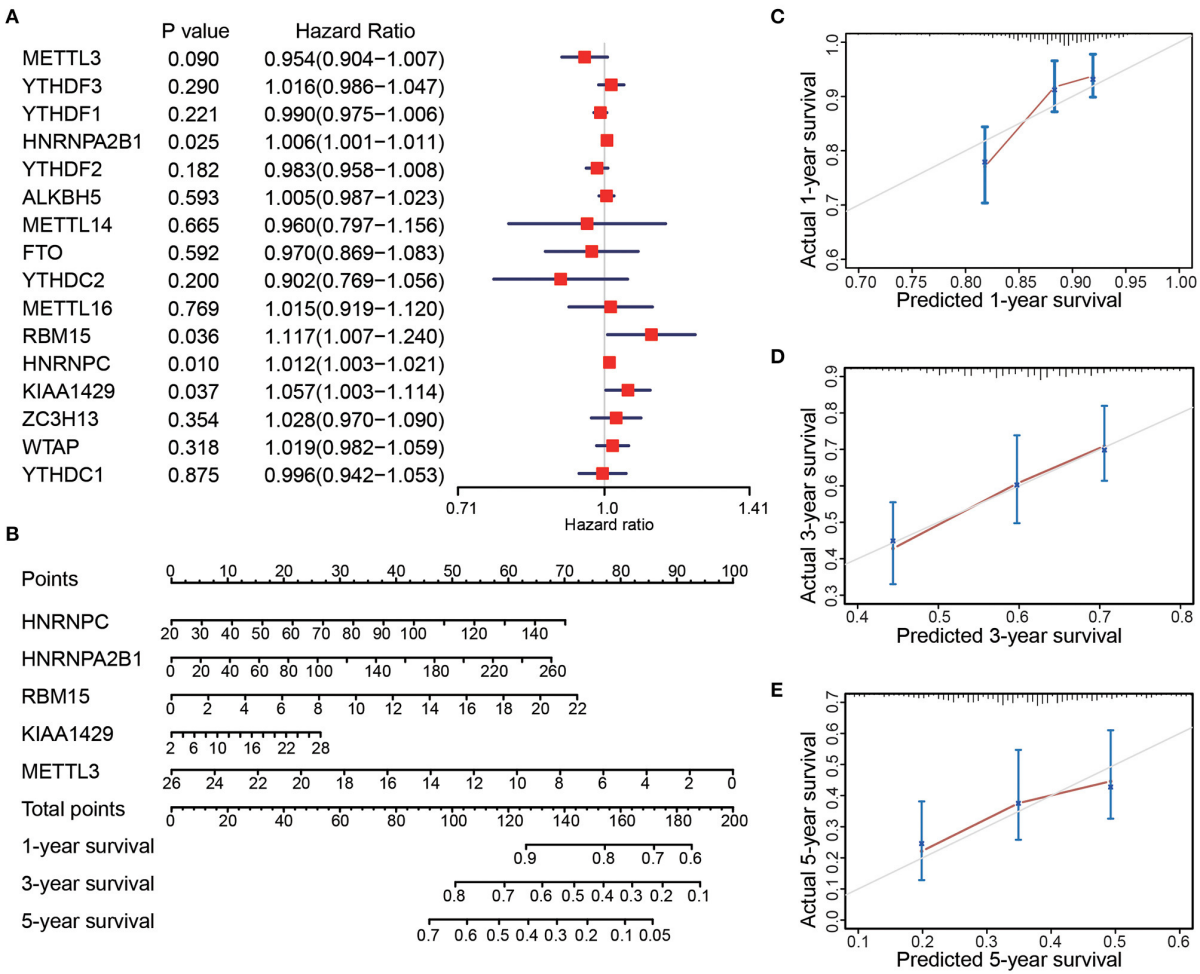
The expression levels of 16 m^6^A-related genes were correlated with the survival time of LUAD patients. **(A)** Forest plot depicting Hazard ratios (HR) and 95% intervals of trust of the m^6^A-related genes. The expression levels of HNRNPA2B1, HNRNPC, KIAA1429, RBM15, and METTL3 were associated to the prognosis of patients. **(B)** Combining the levels of these five m^6^A-related genes to establish a Nomogram chart to predict 1, 3, and 5-year survival patients with LUAD. **(C–E)** Validation curve corresponding to patient 1, 3, and 5-year survival prediction model (Calibrated curve).

Clinical data of the 467 patients were further analyzed to explore the prognostic potential of the expression levels of the m^6^A regulatory factors in LUAD (Table 1). Survival analysis showed that the expression levels of four regulatory factors (HNRNPA2B1, HNRNPC, KIAA1429, and RBM15) were significantly positively associated with the patient's death risk, while METTL3 was negatively associated with it without reaching statistical significance (0.05<p<0.1) (Figure 2A). Then, a Nomogram prediction model based on the expression levels of the above five genes and patient's outcomes data were established (Figure 2B). Four hundred and twenty cases were randomly selected from all cases and divided into three groups with each one of them having 140 cases. The results of the calibration curves indicated that the Nomogram prediction model presented good predictive potential for 1, 3, and 5-year OS of LUAD patients (Figures 2C–E).

**Table 1:** Clinical data of LUAD patients in TCGA database.

	**Features**	**Numbers**	**Percentage %**
Gender	Female	254	54.39
	Male	213	45.61
Age (Y)	65.01 ± 10.05		
	≤65	227	48.61
	>65	240	51.39
Stage	Stage I	251	53.75
	Stage II	108	23.13
	Stage III	75	16.06
	Stage IV	25	5.35
	Unknown	8	1.71
T	T1	162	34.69
	T2	244	52.25
	T3	39	8.35
	T4	19	4.07
	Unknown	3	0.64
M	M0	314	67.24
	M1	24	5.14
	Unknown	129	27.62
N	N0	300	64.24
	N1	87	18.63
	N2	66	14.13
	N3	2	0.43
	Unknown	12	2.57

### LASSO Regression and Risk Co-Efficient

**Figure 3:**
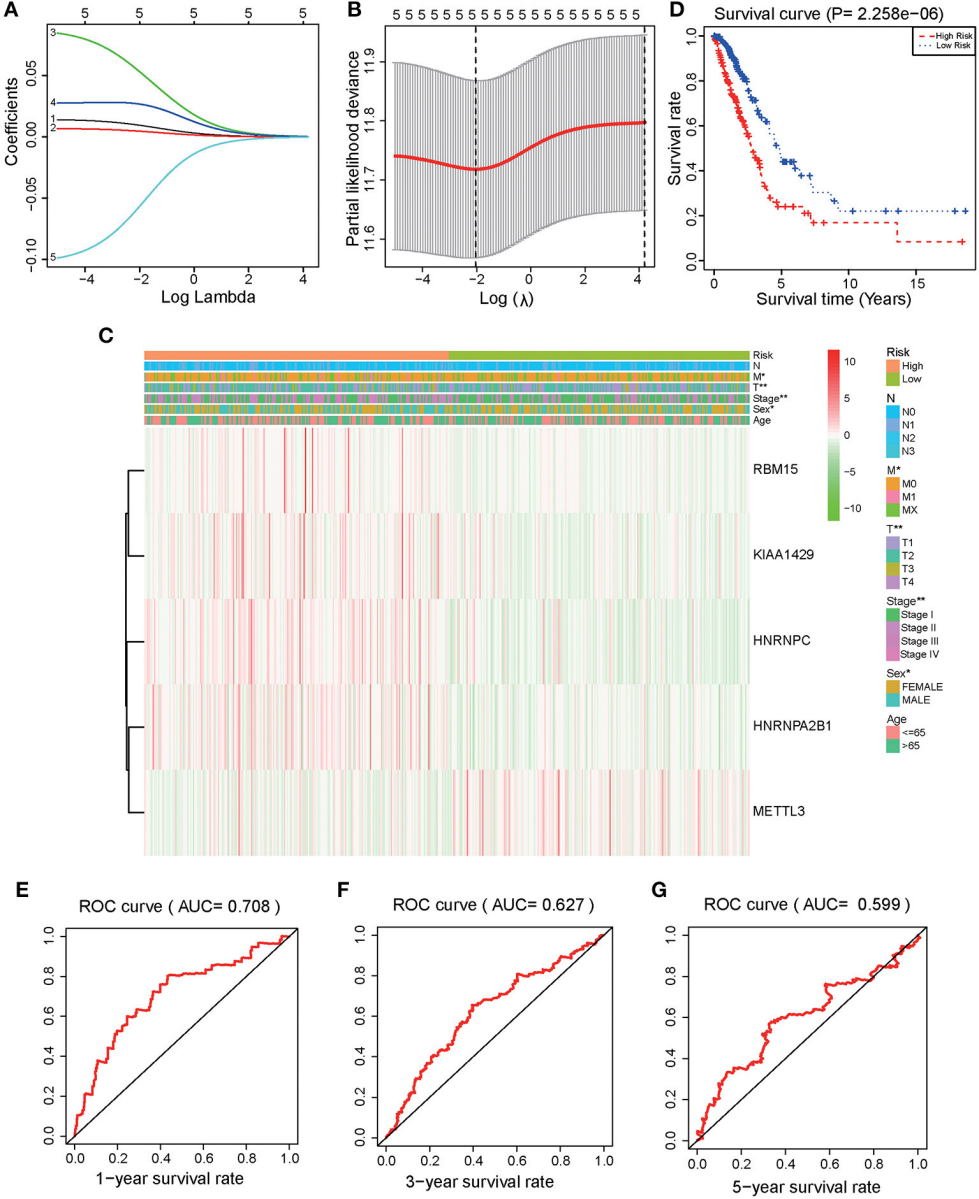
Identification and validation of a risk model to prognose LUAD patients. **(A)** The LASSO analysis model was verified by repeated calculation of the 16 m^6^A genes. **(B)** LASSO coefficient profiles. **(C)** According to LASSO risk factors, LUAD patients were divided into high-risk group and low-risk group. Heatmap demonstrated the expression levels of HNRNPA2B1, HNRNPC, KIAA1429, RBM15, and METTL3 in the two assessed groups. **(D)** Kaplan Meier analysis for the survival of LUAD patients. **(E)** 1-year (*P* < 0.001), **(F)** 3-year (*P* < 0.001), **(G)** 5-year OS ROC curves (*P* < 0.001).

LASSO regression models were used to analyze the risk coefficient and risk value of the expression of the five m^6^A regulators (HNRNPA2B1, HNRNPC, KIAA1429, RBM15, METTL3) in OS prediction (Figures 3A,B). The patients of the present study were divided into high and low-risk groups based on their predicted risk scores. HNRNPA2B1, HNRNPC, KIAA1429, and RBM15 were found to be overexpressed in the high-risk group, while METTL3 was overexpressed in the low-risk group (Figure 3C). Furthermore, survival analysis was conducted using the combined risk value. Results confirmed that the prognosis of patients in the high-risk group was significantly worse than the one in the low-risk group (*P* < 0.01) (Figure 3D). ROC curves for the 1, 3, and 5-year survival prediction demonstrated that the risk value possesses high prognostic accuracy with area under the curve (AUC) of 0.60–0.71 (Figures 3E–G).

### KEGG and Multi-Factor Analysis

**Figure 4:**
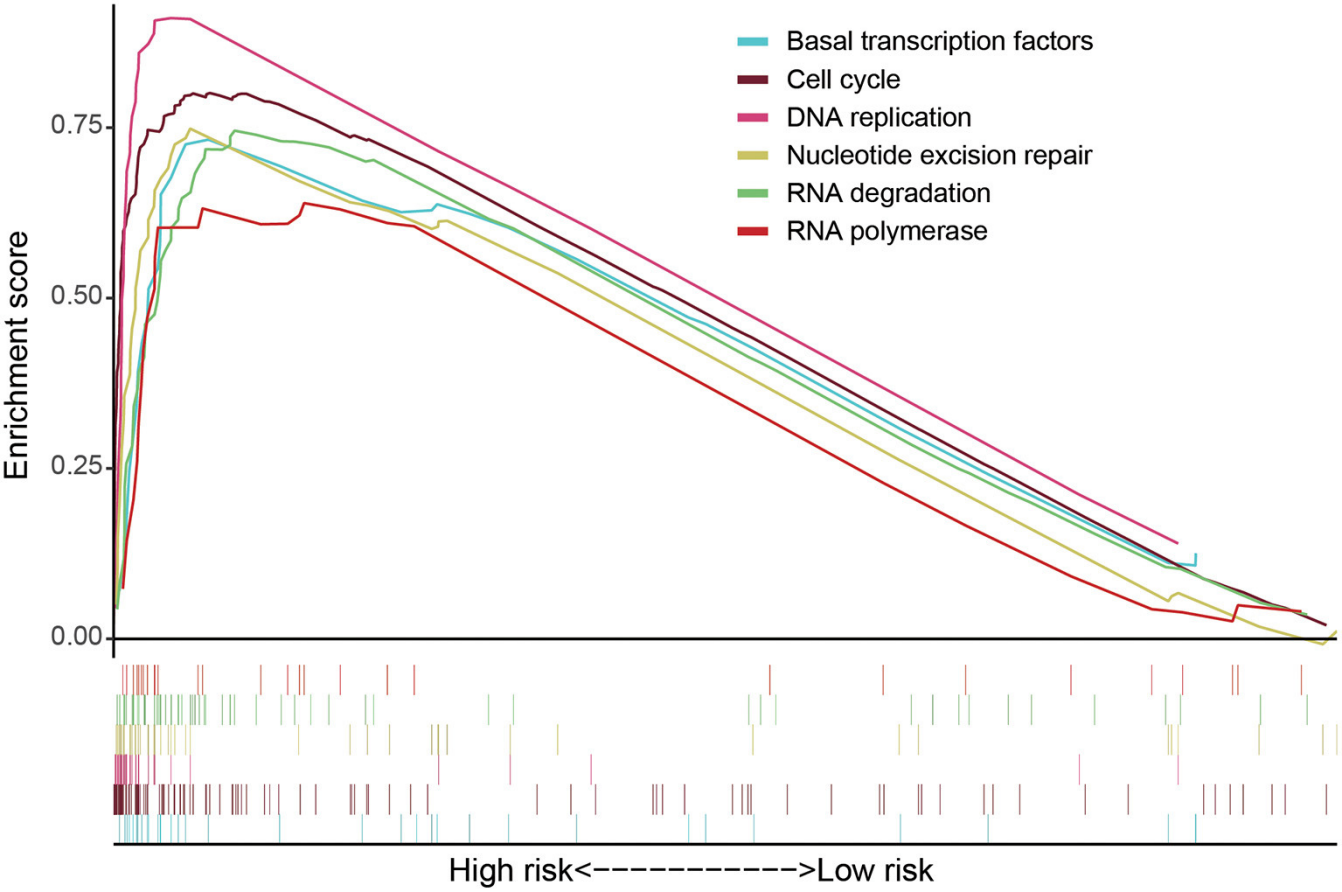
GSEA software was used to analyze the expression differences of internal signal pathways between the two subgroups, including Cell cycle (*P* < 0.001, FDR < 0.001, NES = 2.56), DNA replication (*P* < 0.001, FDR < 0.001, NES = 2.17), RNA degradation (*P* < 0.001, FDR<0.001, NES = 2.49), RNA polymerase (*P* = 0.006, FDR = 0.024, NES = 1.85), Nucleotide excision repair (*P* < 0.001, FDR < 0.001, NES = 2.29), and Basal transcription factors (*P* < 0.001, FDR < 0.001, NES = 2.29) as well as other related signaling pathways (*P* < 0.05, FDR < 0.25).

GSEA software was used to analyze pathway enrichment for the genes that are differentially expressed between high and low-risk patients. The cell pathway was found to be enriched in the set of deregulated factors with a p-value threshold of 0.05 and a FDR threshold of 0.25. The pathway enrichment analysis using KEGG database pathways revealed that Cell cycle (*P* < 0.001, FDR< 0001, Normalized Enrichment Score (NES)=2.56), DNA replication (*P* < 0.001, FDR< 0001, NES = 2.17), RNA degradation (*P* < 0.001, FDR< 0001, NES = 2.49), RNA polymerase (*P* = 0.006, FDR=0.024, NES=1.85), Nucleotide excision repair (*P* < 0.001, FDR< 0001, NES=2.29), Basal transcription factors (*P* < 0.001, FDR < 0001, NES=2.29), and other related signaling pathways are significantly activated in the high-risk group (*P* < 0.05; FDR <0.25) (Figure 4). Accordingly, a heatmap of the most enriched genes for each identified KEGG pathway by GSEA between high and low-risk groups was presented in Supplementary Figures 1–6 and Supplementary Tables 1–6. Univariate and multivariate COX regression analyses were performed based on existing risk factors and patient clinical information (such as age, gender, and tumor and TMN staging) to evaluate their prognostic potential in LUAD (Table 2). The results of univariate analysis suggested that the stage (*P* < 0.001), T stage (*P* < 0.001), lymph node metastasis stage (*P* < 0.001) and risk score (*P* < 0.001) of LUAD patients are significantly negatively associated with the patient's OS. The results of multivariate analysis suggested that the tumor stage (*P* = 0.015), lymph node metastasis stage (*P* = 0.022) and risk score (*P* < 0.001) are significantly negatively associated with the patient's OS.

**Table 2:** Univariate and multivariate COX regression analyses were used to assess the association between the clinical data and risk score of LUAD patients and the prognosis.

	**Univariate COX regression**		**Multivariate COX regression**	
	**Hazard ratio**	***P***	**Hazard ratio**	***P***
Age	1.002 (0.983–1.021)	0.843		
Gender	1.035 (0.717–1.495)	0.852		
Stage	1.654 (1.401–1.951)	<0.001	1.324 (1.056–1.660)	0.015
T	1.632 (1.315–2.024)	<0.001	1.064 (0.836–1.353)	0.615
M	1.757 (0.964–3.203)	0.066		
N	1.790 (1.459–2.196)	<0.001	1.374 (1.046–1.805)	0.022
Risk score	1.793 (1.465–2.195)	<0.001	1.658 (1.331–2.056)	<0.001

**Figure 5:**
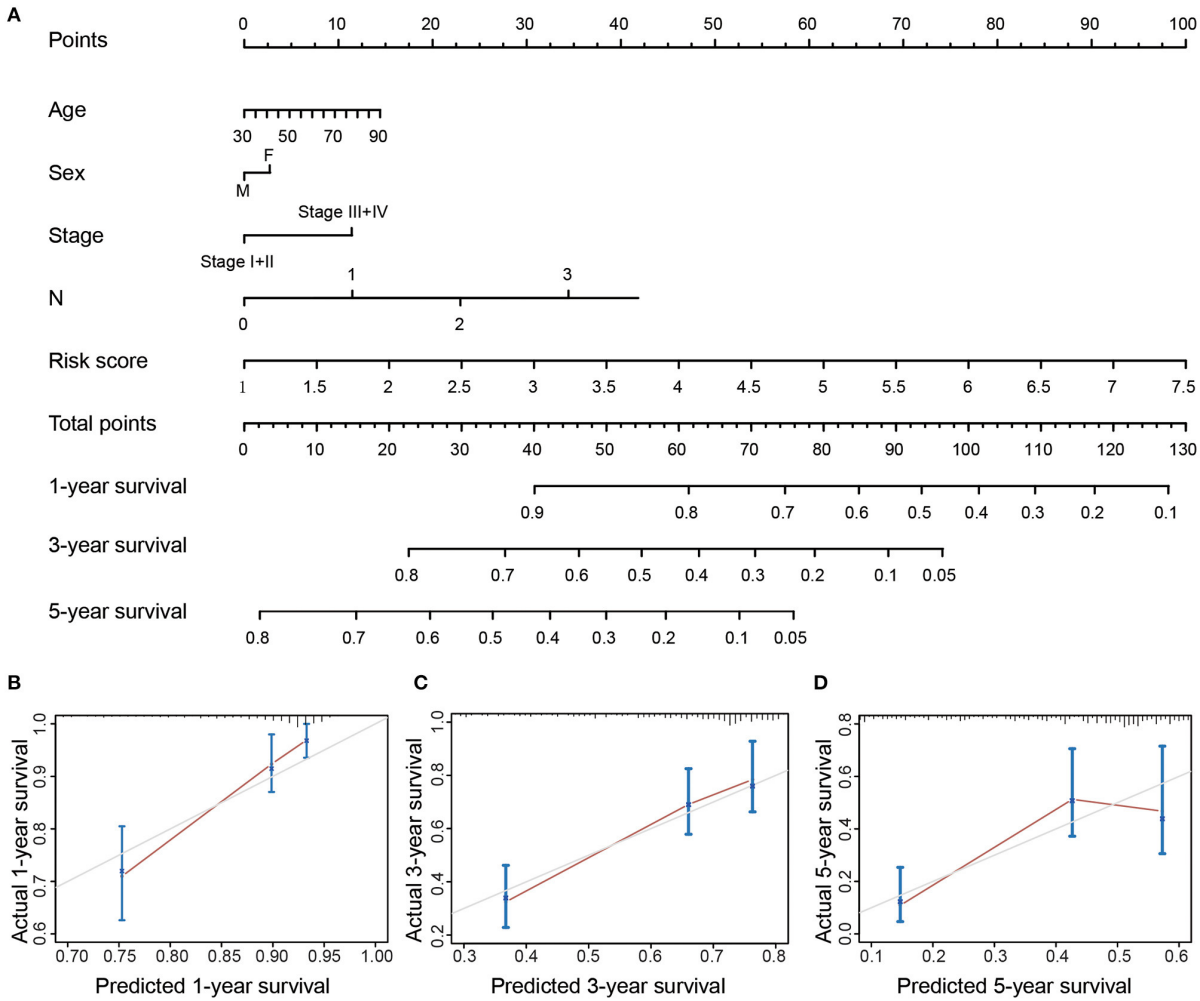
**(A)** Nomogram of OS in LUAD patients. **(B–D)** Corresponding 1/3/5-year survival time verification curve (Calibrated curve).

A Nomogram prognostic analysis model was established based on clinical data such as age, gender, stage, lymph node metastasis and risk value (Figure 5A). The analysis of the 1, 3 and 5-year OS of patients through the Nomogram prediction model has demonstrated that the risk value contributes most to the prediction model followed by lymph node metastasis, age and tumor stage. Three hundred cases were randomly selected from all cases and were then divided into three groups with each one of them having 100 cases. Calibration curves showed that the established Nomogram prediction model presented good predictive potential for the 1, 3, and 5-year OS of LUAD patients (Figures 5B–D).

### m^6^A Writers in Normal Human Organ Tissues

**Figure 6:**
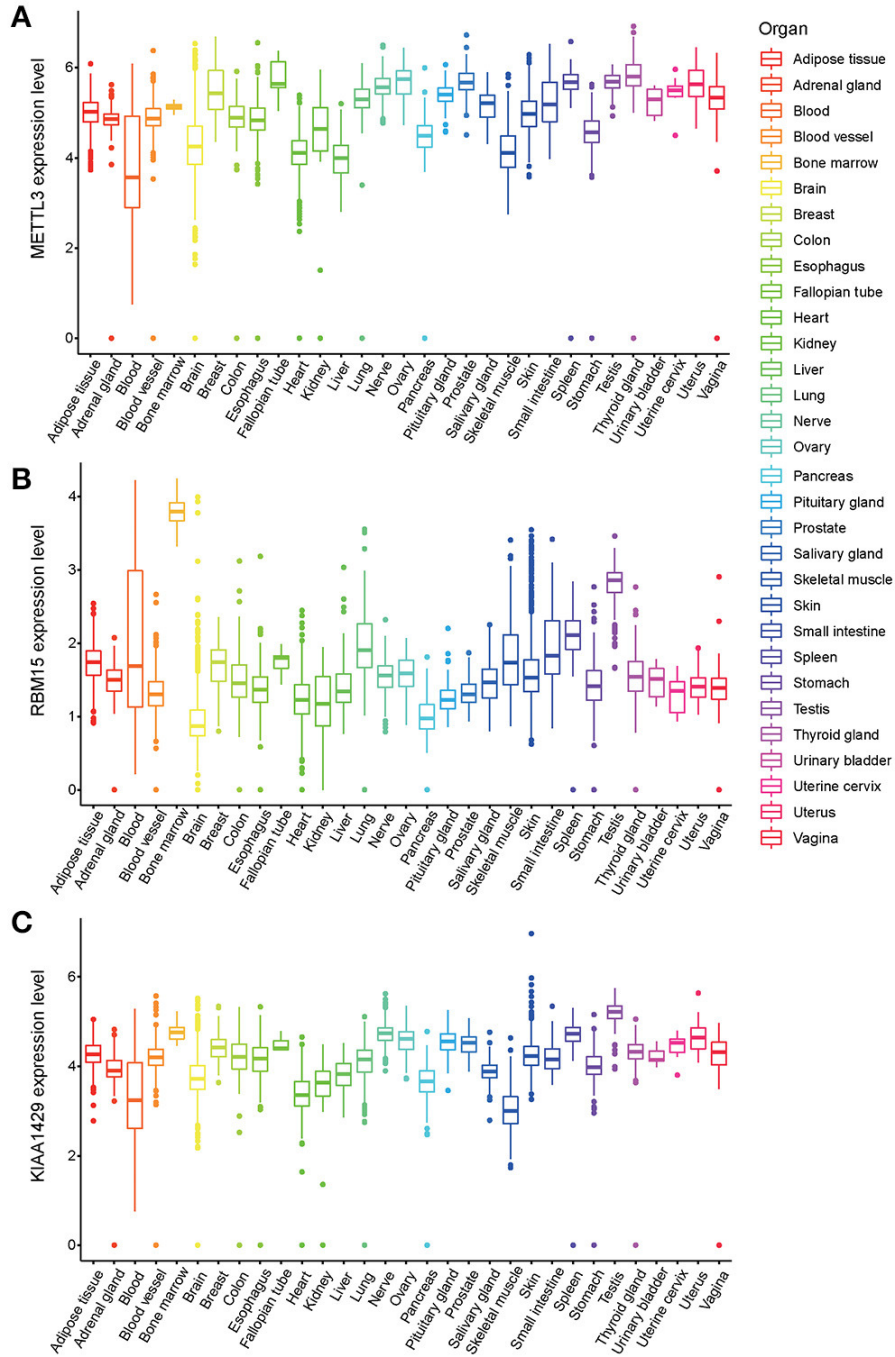
The expression levels of three m^6^A-related genes METTL3 **(A)**, RBM15 **(B)**, and KIAA1429 **(C)** in various organs in the human body.

METTL3, RBM15, and KIAA1429 have been grouped to the m^6^A Writers type (Wang et al., 2018; Chen et al., 2019; Chen and Wong, 2020). LASSO regression analysis showed that the m^6^A Writers, METTL3, RBM15 and KIAA1429, present higher weight co-efficients (Co-ef) (METTL3: −0.0576562669796008; KIAA1429: 0.0269410278179687; RBM15: 0.0539704827385957) than those of HNRNPA2B1 (0.00473147643475602) and HNRNPC (0.00964499370244368). The expression levels of three genes (METTL3, RBM15, and KIAA1429) in 54 human normal organ tissues were compared and analyzed using the GTEx database. No significant difference was found in the comparison between the expression levels of METTL3 and KIAA1429 in various tissues of the human body (Figures 6A,B), while RBM15 was overexpressed in bone marrow and testis compared to other organs in the human body (Figure 6C).

## Discussion

The present study has discussed the m^6^A regulatory factor-related genes are associated to the overall prognosis of LUAD patients, and the newly introduced prognostic model is proven to accurately prognose the outcomes of LUAD patients. Meanwhile, the m^6^A regulatory factor-related genes are associated with the occurrence and development of LUAD.

m^6^A is one of the most common RNA modifications discovered so far. Accumulating studies have recently shown that the deregulation of the m^6^A RNA modification plays an important role in the occurrence and progression of tumors (Cui et al., 2017; Yang et al., 2017; Dai et al., 2018). For instance, the overexpression of FTO in acute myeloid leukemia (AML) can inhibit the m^6^A levels of Ankyrin repeat and SOCS box containing 2 (ASB2) and of retinoic acid receptor α (RARα) mRNA, resulting in the occurrence and progression of AML (Li et al., 2017). The low expression of m^6^A regulators METTL14 in HCC (Ma et al., 2017) and the overexpression of the m^6^A regulator ALKBH5 in glioblastoma (Zhang et al., 2017) have been both associated with poor prognosis. Thus, the abnormal modification of m^6^A is closely related to tumor progress, metastasis and survival prognosis.

The expression levels of a variety of genes involved in RNA methylation mechanisms are closely related to the prognosis of lung cancer patients (Sun et al., 2020). m^6^A methylation is an important RNA modification that occurs in various RNA types such as microRNAs (miRs), circRNAs, and lncRNAs. At the same time, a large number of studies have confirmed that m^6^A and tumor progression are strongly correlated (Ma et al., 2019). The levels of m^6^A-related genes are also tightly associated with the prognosis of lung cancer patients. For instance, Liu and colleagues have confirmed that the expression level of m^6^A is weakly correlated with the prognosis of patients with lung squamous cell carcinoma, while it has a strong correlation with the prognosis of patients with LUAD (Liu et al., 2020). A most recent report from zhuang et al. have reported that the differences in the expression of m^6^A regulators not only have certain diagnostic significance for early lung cancer, but are also closely related to the prognosis of LUAD patients (Zhuang et al., 2020).

In the present study, we have further expanded the number of m^6^A regulatory factors and explored their gene expression levels are related to the prognosis of patients with LUAD. Five m^6^A modification regulators, namely HNRNPA2B1, HNRNPC, KIAA1429, RBM15, and METTL3 are found to be closely related to the prognosis of LUAD patients. LASSO analysis reveals that the outcomes of patients in the high-risk group are significantly worse than the ones in the low-risk group. Both univariate and multivariate analyses conclude that the risk value, stage, and lymph node metastasis are closely related to the patient's prognosis. Finally, a newly developed Nomogram model is able to improve the accuracy of the prognosis of patients compared to conventional risk factors.

The m^6^A writers, METTL3, RBM15, and KIAA1429, were found to be linked with a higher risk in the LASSO regression analysis. Therefore, the abnormal expression of m^6^A writer regulatory factors may affect the prognosis of LUAD. Among them, METTL3 belongs to the class I methyltransferases family and is a predominantly catalytic enzyme in m^6^A modification. METTL3 has been confirmed to be abnormally expressed in a variety of tumors and is believed to be involved in carcinogenesis (Zheng et al., 2019). Prior studies have validated that METTL3 is overexpressed in lung cancer. METTL3 can interact with the transcription factor eIF3h to promote the translation of oncogenes and ultimately to catalyze and accelerate tumor growth and metastasis (Lin et al., 2016; Choe et al., 2018). METTL3 can promote the splicing of the miR-143-3p precursor, which in turn activates the miR-143-3p/VASH1 axis and ultimately leads to the progression and metastasis of lung cancer (Wang et al., 2019). METTL3 has also been shown to increase the expression level of JUNB and promote the occurrence of epithelial-mesenchymal transition (Wanna-Udom et al., 2020). miR-600 (Wei et al., 2019) and miR-33a (Du et al., 2017) is able to inhibit the expression of METTL3, thereby ablating the progression of NSCLC. Thus, METTL3 plays an important role in the occurrence and development of lung cancer. One of the main contributions of the present study is the validation of the hypothesis that the overexpression of METTL3 is negatively correlated with the prognosis of LUAD. This is consistent with the results of previous studies suggesting that the overexpression of METTL3 often indicates poor prognosis in patients with primary liver cancer (Chen et al., 2018).

m^6^A modification regulatory RBM15 is a member of the split end protein (SPEN) family and can bind with METTL3 and WTAP (Wang et al., 2020). Studies have confirmed that m^6^A plays an important regulatory role in the lncRNA XIST-mediated gene transcription silencing. RBM15 catalyzes the recognition of the m^6^A site on lncRNA XIST by METTL3. This m^6^A methylation process can be blocked by the inhibition of RBM15 (Patil et al., 2016). Recent studies have reported that RBM15 assists ZC3H13 in regulating m^6^A methylation, which is essential for speeding up the progress of glioblastoma multiforme (GBM) (Chow et al., 2017; Knuckles et al., 2018). It has been confirmed that RBM15 can regulate the differentiation of megakaryocytes by modulating the alternative splicing of RNA (Jin et al., 2018). Our results have showed that the overexpression of RBM15 may be related to the prognosis of LUAD by affecting m^6^A.

KIAA1429 can upregulate c-Jun mRNA via m^6^A by increasing its stability and by promoting the proliferation of gastric cancer cells (Miao et al., 2020). KIAA1429 increases the expression of cyclin-dependent kinase 1 (CDK1) mRNA to increase the invasion ability of breast cancer cells (Qian et al., 2019). KIAA1429 has been shown to regulate the m^6^A modification of GATA3 precursor mRNA (Lan et al., 2019) and ID2 mRNA (Cheng et al., 2019) in HCC, thereby promoting the progression and metastasis of HCC. In the present study, we have revealed that the expression level of m^6^A Writer KIAA1429 may act as a prognostic marker for LUAD patients.

In conclusion, our findings provide bioinformatics evidence to trigger and support further research on the important role of m^6^A in LUAD. Toward this direction, the validation of the molecular mechanism of m^6^A underlying LUAD occurrence and its association with LUAD prognosis can be further explored by mechanistic experiments with animal models and/or cancer cell lines.

## Data Availability Statement

Requests to access the datasets should be directed to Xiangxuan Zhao, xiangxuanzhao@163.com.

## Author Contributions

XZ and ZL: study design. HW and XZ: data collection. HW, XZ, and ZL: data analysis. XZ and HW: manuscript preparation. All authors read and approved the final manuscript.

## Conflict of Interest

The authors declare that the research was conducted in the absence of any commercial or financial relationships that could be construed as a potential conflict of interest.

## Footnotes

^1^The cancer genome atlas (TCGA) (2020). https://portal.gdc.cancer.gov/
