# Supplementary material for: m6A RNA Methylation Regulators Act as Potential Prognostic Biomarkers in Lung Adenocarcinoma
Source: Front Genet. 2021 Feb 10;12:622233. doi: 10.3389/fgene.2021.622233 (PMC7902930; doi:10.3389/fgene.2021.622233)
Supplement: Supplementary file 1 [file Table_1.DOCX]

| Number | Cell cycle |
| --- | --- |
| 1 | CCNB1 |
| 2 | CCNA2 |
| 3 | MAD2L1 |
| 4 | BIIB1 |
| 5 | TTK |
| 6 | CDC25C |
| 7 | BIIB1B |
| 8 | PLK1 |
| 9 | BIIB3 |
| 10 | CDC20 |
| 11 | DBF4 |
| 12 | CCNB2 |
| 13 | CDC25A |
| 14 | ORC1 |
| 15 | CDK1 |
| 16 | CDC27 |
| 17 | MCM6 |
| 18 | MCM4 |
| 19 | CHEK1 |
| 20 | CDC7 |
| 21 | ESPL1 |
| 22 | RBL1 |
| 23 | YWHAQ |
| 24 | MCM2 |
| 25 | CDC6 |
| 26 | SMC3 |
| 27 | PRKDC |
| 28 | CDC45 |
| 29 | PCNA |
| 30 | CDK2 |
| 31 | E2F3 |
| 32 | PTTG1 |
| 33 | YWHAZ |
| 34 | MCM3 |
| 35 | HDAC2 |
| 36 | ANAPC1 |
| 37 | YWHAG |
| 38 | ORC6 |
| 39 | PKMYT1 |
| 40 | MCM7 |
| 41 | E2F2 |
| 42 | CCNE2 |
| 43 | RAD21 |

| Number | Cell cycle |
| --- | --- |
| 44 | YWHAB |
| 45 | MCM5 |
| 46 | E2F1 |
| 47 | MYC |
| 48 | ORC5 |
| 49 | GSK3B |
| 50 | SMC1A |
| 51 | CHEK2 |
| 52 | SKP2 |
| 53 | CCNE1 |
| 54 | ANAPC7 |
| 55 | ANAPC10 |
| 56 | STAG1 |
| 57 | YWHAE |
| 58 | CDK6 |
| 59 | MAD2L2 |
| 60 | ORC2 |
| 61 | CDC23 |
| 62 | ORC3 |
| 63 | ORC4 |
| 64 | STAG2 |
| 65 | CDK7 |
| 66 | ATR |
| 67 | CDKN2A |
| 68 | CDKN2C |
| 69 | SMAD2 |
| 70 | HDAC1 |
| 71 | CDC26 |
| 72 | ANAPC5 |
| 73 | CIIL1 |
| 74 | RB1 |
| 75 | CCNA1 |
| 76 | ANAPC13 |
| 77 | SMC1B |
| 78 | CCNB3 |
| 79 | CDKN2D |
| 80 | GADD45A |
| 81 | CDK4 |
| 82 | E2F5 |
| 83 | WEE1 |
| 84 | TFDP1 |
| 85 | SFN |
| 86 | ANAPC11 |

| Number | Cell cycle |
| --- | --- |
| 87 | CDC25B |
| 88 | TFDP2 |
| 89 | YWHAH |
| 90 | PTTG2 |
| 91 | ABL1 |
| 92 | CCNH |
| 93 | SMAD4 |
| 94 | E2F4 |
| 95 | CDKN1B |
| 96 | MDM2 |
| 97 | EP300 |
| 98 | TGFB2 |
| 99 | SKP1 |
| 100 | MAD1L1 |
| 101 | CDKN1C |
| 102 | CDKN2B |
| 103 | SMAD3 |
| 104 | CDC14A |
| 105 | TGFB3 |
| 106 | ATM |
| 107 | WEE2 |
| 108 | ANAPC4 |
| 109 | CDKN1A |
| 110 | RBX1 |
| 111 | CCND1 |
| 112 | CDC14B |
| 113 | CCND2 |
| 114 | TP53 |
| 115 | CREBBP |
| 116 | CDC16 |
| 117 | FZR1 |
| 118 | GADD45B |
| 119 | TGFB1 |
| 120 | CCND3 |
| 121 | ZBTB17 |
| 122 | GADD45G |
| 123 | ANAPC2 |
| 124 | RBL2 |
