# Supplementary material for: m6A RNA Methylation Regulators Act as Potential Prognostic Biomarkers in Lung Adenocarcinoma
Source: Front Genet. 2021 Feb 10;12:622233. doi: 10.3389/fgene.2021.622233 (PMC7902930; doi:10.3389/fgene.2021.622233)
Supplement: Supplementary file 2 [file Table_2.DOCX]

| Number | DNA replication |
| --- | --- |
| 1 | MCM6 |
| 2 | FEN1 |
| 3 | MCM4 |
| 4 | RFC5 |
| 5 | RFC3 |
| 6 | MCM2 |
| 7 | RNASEH1 |
| 8 | PCNA |
| 9 | PRTM1 |
| 10 | PRTM2 |
| 11 | MCM3 |
| 12 | POLE3 |
| 13 | POLA2 |
| 14 | RNASEH2A |
| 15 | RFC4 |
| 16 | POLE2 |
| 17 | DNA2 |
| 18 | POLA1 |
| 19 | MCM7 |
| 20 | RFC2 |
| 21 | POLD3 |
| 22 | RPA3 |
| 23 | RFC1 |
| 24 | MCM5 |
| 25 | POLD2 |
| 26 | SSBP1 |
| 27 | POLE |
| 28 | RPA1 |
| 29 | LIG1 |
| 30 | POLE4 |
| 31 | POLD1 |
| 32 | RPA2 |
| 33 | RPA4 |
| 34 | RNASEH2B |
| 35 | RNASEH2C |
| 36 | POLD4 |
