# Supplementary material for: m6A RNA Methylation Regulators Act as Potential Prognostic Biomarkers in Lung Adenocarcinoma
Source: Front Genet. 2021 Feb 10;12:622233. doi: 10.3389/fgene.2021.622233 (PMC7902930; doi:10.3389/fgene.2021.622233)
Supplement: Supplementary file 3 [file Table_3.DOCX]

| Number | RNA degradation |
| --- | --- |
| 1 | HSPD1 |
| 2 | CNOT9 |
| 3 | EXOSC3 |
| 4 | PNPT1 |
| 5 | LSM5 |
| 6 | EXOSC2 |
| 7 | PATL1 |
| 8 | EXOSC9 |
| 9 | CNOT10 |
| 10 | LSM6 |
| 11 | HSPA9 |
| 12 | PAPOLA |
| 13 | XRN2 |
| 14 | ZCCHC7 |
| 15 | ENO1 |
| 16 | PAPOLG |
| 17 | DTS3 |
| 18 | LSM3 |
| 19 | CNOT6 |
| 20 | LSM2 |
| 21 | CNOT1 |
| 22 | C1D |
| 23 | MPHOSPH6 |
| 24 | EDC3 |
| 25 | DCP1A |
| 26 | XRN1 |
| 27 | MTREX |
| 28 | LSM1 |
| 29 | EXOSC10 |
| 30 | LSM8 |
| 31 | EXOSC8 |
| 32 | EXOSC5 |
| 33 | DDX6 |
| 34 | CNOT7 |
| 35 | DCPS |
| 36 | LSM4 |
| 37 | CNOT6L |
| 38 | CNOT4 |
| 39 | EXOSC4 |
| 40 | EXOSC1 |
| 41 | AC092881_1 |
| 42 | TTC37 |

| Number | RNA degradation |
| --- | --- |
| 43 | DCP2 |
| 44 | PAPOLB |
| 45 | CNOT3 |
| 46 | C1DP3 |
| 47 | ENO2 |
| 48 | ENO3 |
| 49 | TENT4A |
| 50 | LSM7 |
| 51 | EXOSC7 |
| 52 | EXOSC6 |
| 53 | C1DP2 |
| 54 | SKTV2L |
| 55 | WDR61 |
| 56 | EDC4 |
| 57 | CNOT8 |
| 58 | PARN |
| 59 | DCP1B |
