# Supplementary material for: m6A RNA Methylation Regulators Act as Potential Prognostic Biomarkers in Lung Adenocarcinoma
Source: Front Genet. 2021 Feb 10;12:622233. doi: 10.3389/fgene.2021.622233 (PMC7902930; doi:10.3389/fgene.2021.622233)
Supplement: Supplementary file 4 [file Table_4.DOCX]

| Number | RNA polymerase |
| --- | --- |
| 1 | POLR1B |
| 2 | POLR2D |
| 3 | POLR2B |
| 4 | POLR3G |
| 5 | POLR1A |
| 6 | POLR3A |
| 7 | POLR1E |
| 8 | POLR3C |
| 9 | POLR3B |
| 10 | POLR3F |
| 11 | POLA2K |
| 12 | POLR2H |
| 13 | POLR2G |
| 14 | ZNRD1 |
| 15 | POLR3D |
| 16 | POLR3K |
| 17 | POLR2I |
| 18 | POLR2J |
| 19 | POLR1C |
| 20 | POLR2A |
| 21 | POLR1D |
| 22 | POLR2J3 |
| 23 | POLR2E |
| 24 | POLR2J2 |
| 25 | POLR2C |
| 26 | POLR2F |
| 27 | POLR2L |
| 28 | POLR3H |
| 29 | POLR3GL |
