# Supplementary material for: m6A RNA Methylation Regulators Act as Potential Prognostic Biomarkers in Lung Adenocarcinoma
Source: Front Genet. 2021 Feb 10;12:622233. doi: 10.3389/fgene.2021.622233 (PMC7902930; doi:10.3389/fgene.2021.622233)
Supplement: Supplementary file 5 [file Table_5.DOCX]

| Number | Nucleotide excision repair |
| --- | --- |
| 1 | RAD23B |
| 2 | RFC5 |
| 3 | RFC3 |
| 4 | GTF2H3 |
| 5 | PCNA |
| 6 | POLE3 |
| 7 | RFC4 |
| 8 | POLE2 |
| 9 | RFC2 |
| 10 | POLD3 |
| 11 | RPA3 |
| 12 | ERCC8 |
| 13 | RFC1 |
| 14 | POLD2 |
| 15 | CIIL4B |
| 16 | DDB1 |
| 17 | POLE |
| 18 | RPA1 |
| 19 | LIG1 |
| 20 | GTF2H1 |
| 21 | MNAT1 |
| 22 | POLE4 |
| 23 | CDK7 |
| 24 | RAD23A |
| 25 | POLD1 |
| 26 | ERCC4 |
| 27 | ERCC6 |
| 28 | ERCC3 |
| 29 | CCNH |
| 30 | GTF2H2 |
| 31 | GTF2H5 |
| 32 | RPA2 |
| 33 | CIIL4A |
| 34 | RPA4 |
| 35 | ERCC2 |
| 36 | ERCC1 |
| 37 | CETN2 |
| 38 | RBX1 |
| 39 | GTF2H4 |
| 40 | XPA |
| 41 | POLD4 |
| 42 | DDB2 |

| Number | Nucleotide excision repair |
| --- | --- |
| 43 | ERCC5 |
| 44 | XPC |
