# Supplementary material for: m6A RNA Methylation Regulators Act as Potential Prognostic Biomarkers in Lung Adenocarcinoma
Source: Front Genet. 2021 Feb 10;12:622233. doi: 10.3389/fgene.2021.622233 (PMC7902930; doi:10.3389/fgene.2021.622233)
Supplement: Supplementary file 6 [file Table_6.DOCX]

| Number | Basal transcription factors |
| --- | --- |
| 1 | GTF2H3 |
| 2 | GTF2E1 |
| 3 | GTF2A1 |
| 4 | TAF2 |
| 5 | TAF5 |
| 6 | TAF5L |
| 7 | TAF13 |
| 8 | GTF2F2 |
| 9 | TAF9 |
| 10 | GTF2B |
| 11 | TAF11 |
| 12 | GTF2E2 |
| 13 | GTF2A2 |
| 14 | GTF2H1 |
| 15 | TAF4B |
| 16 | TAF6 |
| 17 | TBP |
| 18 | TBPL1 |
| 19 | GTF2A1L |
| 20 | GTF2TRD1 |
| 21 | STON1 |
| 22 | TAF9B |
| 23 | GTF2H2 |
| 24 | TAF7L |
| 25 | TAF4 |
| 26 | TAF12 |
| 27 | TAF7 |
| 28 | GTF2F1 |
| 29 | GTF2T |
| 30 | TAF10 |
| 31 | TAF1 |
| 32 | TBPL2 |
| 33 | GTF2H4 |
| 34 | TAF6L |
| 35 | TAF1L |
