# Supplementary material for: m6A RNA Methylation Regulators Act as Potential Prognostic Biomarkers in Lung Adenocarcinoma
Source: Front Genet. 2021 Feb 10;12:622233. doi: 10.3389/fgene.2021.622233 (PMC7902930; doi:10.3389/fgene.2021.622233)
Supplement: Supplementary file 7 [file Data_Sheet_1.DOCX]

**Supplementary Figure and Table legends**

**Figure S1‒6. A heatmap of the most enriched genes for each identified KEGG pathway by GSEA between high and low-risk groups.** S1. Cell cycle; S2. DNA replication; S3. RNA degradation; S4. RNA polymerase; S5. Nucleotide excision repair; S6. Basal transcription factors. (High score group: gray color; Low score group: orange color)

**Table S1‒6**. **Enriched genes for each identified KEGG pathway.** S1. Cell cycle; S2. DNA replication; S3. RNA degradation; S4. RNA polymerase; S5. Nucleotide excision repair; S6. Basal transcription factors.
